# Menopausal hormone therapy, oral contraceptives and risk of chronic low back pain: the HUNT Study

**DOI:** 10.1186/s12891-023-06184-5

**Published:** 2023-01-31

**Authors:** Ingrid Heuch, Ivar Heuch, Knut Hagen, Kjersti Storheim, John-Anker Zwart

**Affiliations:** 1 Department of Research and Innovation, Division of Clinical Neuroscience, Oslo University Hospital, Nydalen, P.O. Box 4956, N-0424 Oslo, Norway; 2 Department of Mathematics, University of Bergen, Bergen, Norway; 3 Department of Neuromedicine and Movement Science, NTNU, Norwegian University of Science and Technology, Trondheim, Norway; 4 Clinical Research Unit Central Norway, St. Olavs Hospital, Trondheim, Norway; 5 Faculty of Health Sciences, Oslo Metropolitan University, Oslo, Norway; 6 Faculty of Medicine, University of Oslo, Oslo, Norway

**Keywords:** Epidemiology, Hormone replacement therapy, HUNT, Low back pain, Oestrogen, Oral contraceptives

## Abstract

**Background:**

There are indications that use of menopausal hormone therapy (MHT) and oral contraceptives (OC) increases the risk of low back pain (LBP), with higher oestrogen levels involved in the underlying mechanisms. The purpose of the present study was to investigate associations between use of systemic MHT or OC and risk of chronic LBP in a large population-based data set.

**Methods:**

Data were obtained from two surveys in the Trøndelag Health Study in Norway, HUNT2 (1995–1997) and HUNT3 (2006–2008). A cross-sectional study of association between use of systemic MHT and prevalence of chronic LBP comprised 12,974 women aged 40–69 years in HUNT2, with 4007 women reporting chronic LBP. A cohort study involving MHT comprised 6007 women without chronic LBP at baseline in HUNT2, and after 11 years 1245 women reported chronic LBP at follow-up in HUNT3. The cross-sectional study of association with use of OC included 23,593 women aged 20–69 years in HUNT2, with 6085 women reporting chronic LBP. The corresponding cohort study included 10,586 women without chronic LBP at baseline in HUNT2, of whom 2084 women reported chronic LBP in HUNT3. Risk of chronic LBP was examined in both study designs in generalised linear models with adjustment for potential confounders.

**Results:**

In the cohort study, current users of systemic MHT at baseline showed a greater risk of chronic LBP (relative risk (RR) 1.30; 95% CI: 1.14–1.49; compared with never users). The risk increased according to duration of MHT use (*P* for linear trend = 0.003). Known users of systemic MHT based exclusively on oestrogen experienced the highest risk (RR 1.49; 95% CI: 1.16–1.91), but an increased risk was also seen among known users of oestrogen-progestin combination MHT (RR 1.35; 95% CI: 1.16–1.57). A slight increase in risk of chronic LBP was found in the cohort study among former users of OC (RR 1.17; 95% CI: 1.06–1.30; compared with never users).

**Conclusions:**

Long-lasting use of systemic MHT, in particular therapy based on oestrogen only, is associated with greater risk of chronic LBP. Having been a user of OC most likely entails a minor increase in risk.

**Supplementary Information:**

The online version contains supplementary material available at 10.1186/s12891-023-06184-5.

## Background

Low back pain (LBP) represents a serious disorder, both for the individuals affected and for society [1]. The consequences in terms of lost working hours and expenses incurred are far-reaching [2]. It has been hypothesised that the risk of LBP in women is influenced by hormonal levels [3]. Thus, potential relationships with parity have been explained by changes in oestrogen levels during pregnancy [4], and an association with age at menarche may also reflect differential exposure to oestrogen [3]. Moreover, a hormonal influence may be responsible for the age dependence of the prevalence ratio of LBP comparing women and men [5].

There are indications that menopausal hormone therapy (MHT) can increase the risk of LBP [6]. This hypothesis has been supported by results from large population-based studies of LBP [3] or back pain more generally [7], although earlier smaller studies [8, 9] produced more ambiguous results. In contrast, a controlled trial among slim osteopenic women found a preventive effect of oestrogen-progestin treatment on back pain [10].

Use of oral contraceptives (OC) has been widely regarded as a risk factor for LBP [11], but the evidence is scarce. A few relatively large studies have shown moderate positive associations between OC use and risk or prevalence of LBP [3, 12] or more general back pain [9, 13], although other studies were unable to confirm these results [6, 8, 14, 15]. Studies of back pain during or after pregnancy have either found no association with earlier OC use [16] or an inverse relationship [17].

Relationships in women between prevalence of chronic LBP and parity or age at pregnancies have previously been explored in a large Norwegian population-based data set [18]. An increased prevalence was found among women with a first childbirth before the age of 20 years. An early menarche at age ≤ 11 years was also associated with a higher risk of chronic LBP [19]. The purpose of the present study was to investigate associations between use of systemic MHT or OC and prevalence or risk of chronic LBP in the same data set, taking into account the effects of relevant confounders.

## Methods

### Collection of information

As part of the Trøndelag Health Study, the survey HUNT2 was carried out in Norway in 1995–1997, and the survey HUNT3 was conducted approximately 11 years later in 2006–2008 [20, 21]. All residents at the time in the former Nord-Trøndelag county aged ≥ 20 years were invited to participate in each survey. They were requested to fill in questionnaires on health status and to take part in clinical consultations, which included measurement of height and weight.

One question in the HUNT2 and HUNT3 questionnaires was formulated in this way: “During the last year, have you suffered from pain and/or stiffness in your muscles and joints that has lasted for at least 3 consecutive months?ˮ Each participant answering yes was given the following question: “Where did you have these complaints?” Several body regions were listed. Respondents answering yes to the first question and including the lower back as a relevant region were regarded as having chronic LBP [22].

In HUNT2 the participants gave information about use of MHT by answering this question: “Have you ever used medicines containing oestrogen?” Examples of common brand names were displayed, and it was emphasised that the question did not refer to OC use. The questionnaire distinguished between use of tablets or patches and use of cream or suppositories. In each case, the respondents were requested to indicate whether they had now, previously or never been engaged in the kind of use considered and to specify duration of usage. Finally, current users were requested to supply the particular brand name of the product. Use of tablets or patches was regarded as systemic MHT while cream or suppositories represented local use. The proportion of women providing information about possible local use was much lower than for systemic use, and only systemic use is considered in the present study. On the basis of brand names, current systemic MHT use was either classified as containing oestrogen only or as representing a combination of oestrogen and progestin.

The women provided information about use of OC by answering the following question: ”Have you ever taken contraceptive pills, including mini-pills?” Women who had ever used OC were then asked whether they were still OC users. They were also requested to indicate the duration of OC use.

Women participating in HUNT2 gave information on age at menarche by answering the question “How old were you when you started menstruating?” The participants also gave information regarding physical activity in leisure time, smoking, duration of education and childbirths. In addition, they provided information used for computing Hospital Anxiety and Depression Scale (HADS) scores [23].

### Study design

#### Use of MHT

**Fig. 1:**
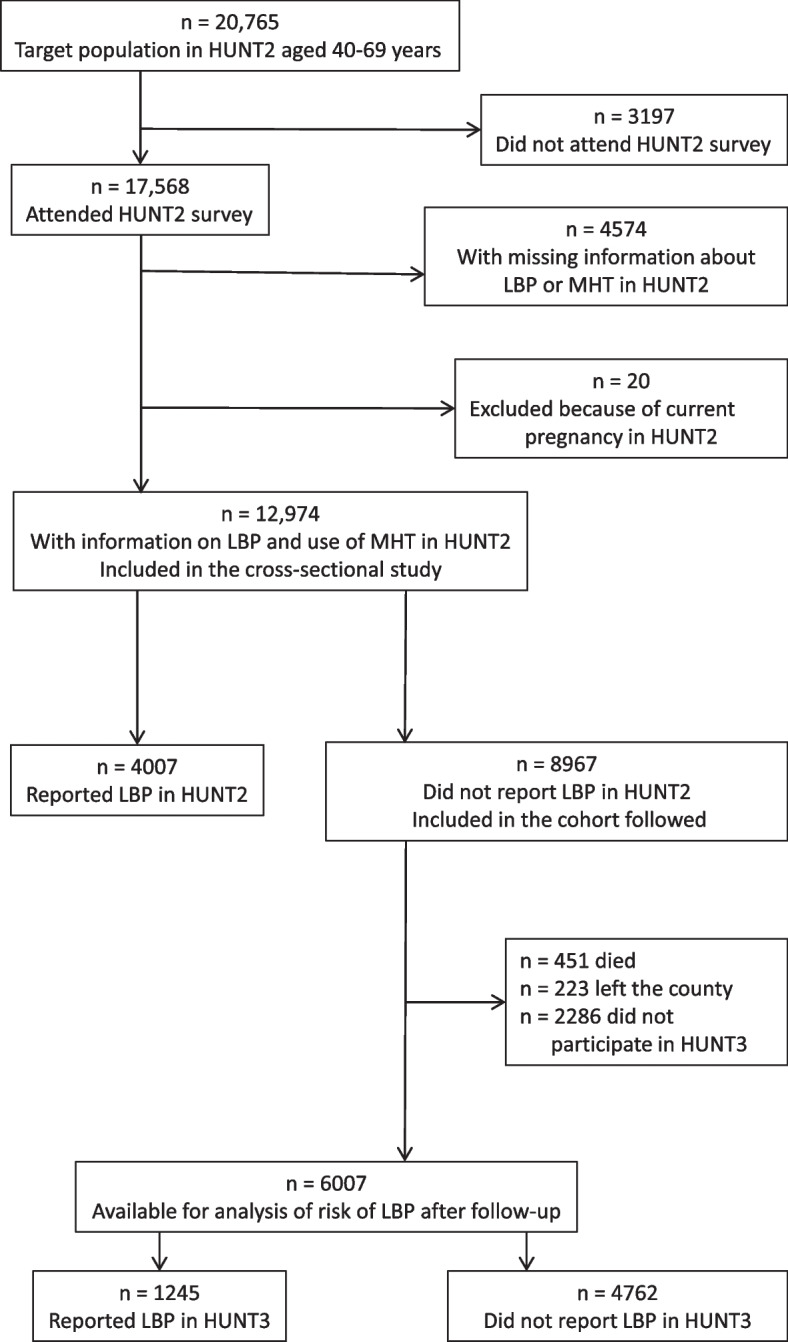
Flow chart for the cross-sectional and cohort studies of associations between MHT and LBP. HUNT, Trøndelag Health Study; LBP, low back pain; MHT, menopausal hormone therapy

The study of associations with MHT was restricted to women in the age range 40–69 years. The corresponding target population in HUNT2 comprised 20,765 women. Of these, 17,568 attended the HUNT2 survey (Fig. 1). A total of 4574 women were excluded from this study because of missing information on chronic LBP or MHT, and 20 women were excluded because they were pregnant when the survey was carried out. Information on chronic LBP and use of MHT in HUNT2 was collected from the remaining 12,974 women included in the cross-sectional study, corresponding to a participation rate of 62%.

The 8967 women who did not report chronic LBP in HUNT2 were included in the 11-year follow-up (Fig. 1). Information about residence status was collected from national registries and linked by the unique personal identification numbers being used in Norway. During follow-up 451 women died, and 223 left the county. A total of 2286 women who lived in the county at the time of HUNT3 did not participate in HUNT3 or did not supply information on chronic LBP. Thus, 6007 women were included in the analysis of risk of chronic LBP after follow-up, representing 72% of the women remaining in the county and 67% of the original cohort.

#### Use of OC

**Fig. 2:**
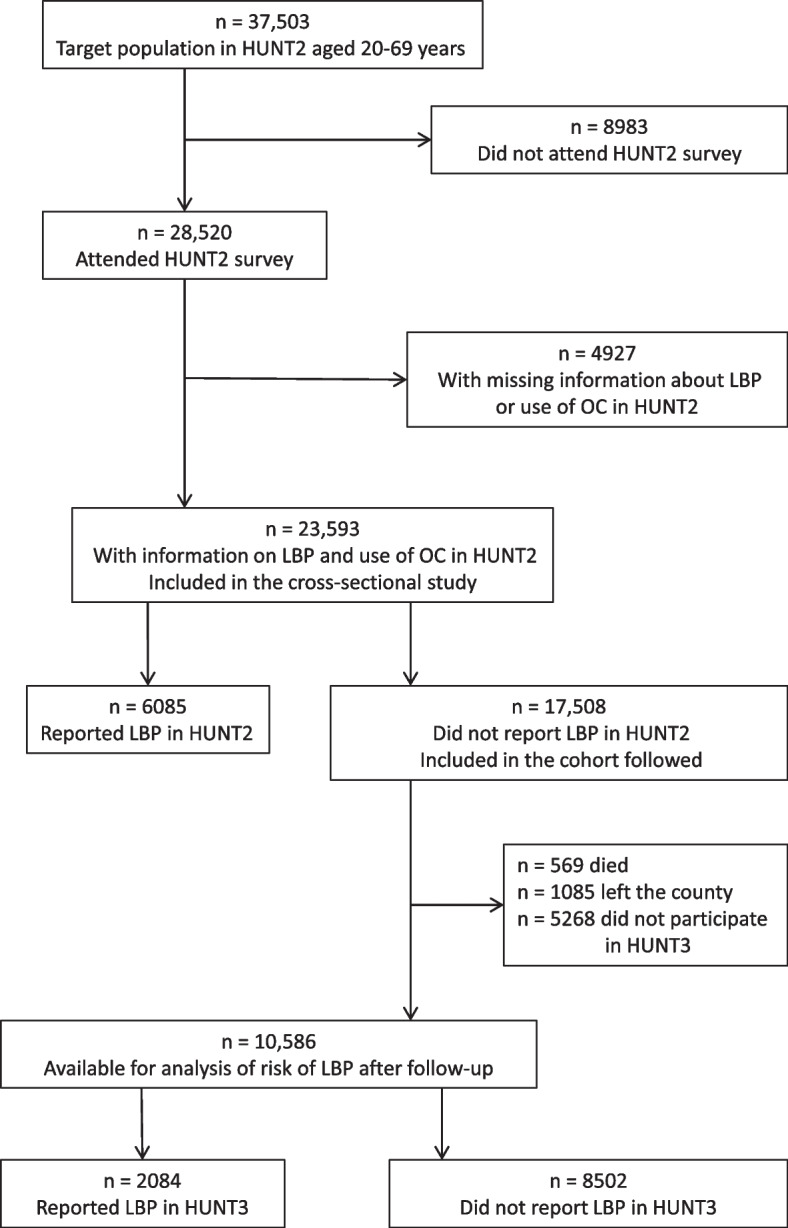
Flow chart for the cross-sectional and cohort studies of associations between OC and LBP. HUNT, Trøndelag Health Study; LBP, low back pain; OC, oral contraceptives

The target population in HUNT2 comprised 37,503 women in the age interval 20–69 years. Of these, a total of 28,520 women attended the HUNT2 survey (Fig. 2). Information on chronic LBP and use of OC in HUNT2 was collected from 23,593 women, corresponding to a participation rate of 63%. This data set formed the basis of the cross-sectional study of associations with use of OC.

The subset of 17,508 women who did not report chronic LBP in HUNT2 were included in the cohort study (Fig. 2). During the period of follow-up, 569 women died and 1085 left the county. A total of 5268 women did not participate or did not supply information on chronic LBP. Altogether 10,586 women were available for analysis of risk of chronic LBP after follow-up, representing 67% of the remaining women resident in the county and 60% of the original cohort.

### Variables

Use of systemic MHT as reported in HUNT2 was considered in 3 categories as never, former and current use. Duration of systemic MHT use included 5 categories corresponding to never use, 1 month-2 years, 3–5 years, 6–8 years and ≥ 9 years. For type of systemic MHT among current users, categories represented therapy based on oestrogen only or combination therapy, in addition to never use. Use of OC was also categorised as never, former and current use. Duration of OC use included 5 categories, corresponding to never use, 1 month-4 years, 5–9 years, 10–14 years and ≥ 15 years. In all situations, never use was regarded as reference category.

Body mass index (BMI), defined as weight/height^2^ and computed in kg/m^2^, was subdivided into 3 groups: < 25, 25–29.9, ≥ 30. For physical activity in leisure time, including going to work, the first category represented those engaged in light activity only or hard physical activity (leading to sweating or being out of breath) < 1 h per week. Other categories represented hard physical activity 1–2 and ≥ 3 h per week. The information about physical activity collected in HUNT2 was verified by a reliability and validity study of a subsample [24]. Education was grouped according to duration as ≤ 9, 10–12 and ≥ 13 years. Categories of cigarette smoking represented current daily smoking, previous daily smoking and never daily smoking.

Age at menarche was categorised into 7 groups: ≤ 11, 12, 13, 14, 15, 16, ≥ 17 years. A particular variable was introduced to take into account both nulliparity and age at first childbirth (in 5-year categories) among parous women. Women who were pregnant at the time of HUNT2 constituted a separate category. Total HADS scores were categorised into 5 intervals, 0–4, 5–9, 10–14, 15–19 and ≥ 20, to obtain a relatively detailed representation of psychological factors.

### Statistical analysis

Relationships in the data set analysed between use of systemic MHT or OC and other potential risk factors were described in an exploratory approach by tabulating mean values or frequency distributions over categories of MHT and OC. To assess the extent of differential participation at end of follow-up, similar tabulations were performed among the women who were residents of Nord-Trøndelag at the time of follow-up but did not participate, and among those who moved out of the county or died during follow-up.

Generalised linear modelling for binomial observations with a log link was applied to both cross-sectional and cohort data to study associations between use of MHT or OC and chronic LBP. The initial analysis incorporated adjustment for age only. Additional adjustment was then introduced for other relevant factors known to be risk factors for LBP and suspected to be associated with use of MHT or OC. This involved BMI [25–27], physical activity in leisure time [28–30], education [31–33] and smoking [34–36]. In view of the associations established previously in this data set [18, 19], adjustment was also made for age at menarche, nulliparity and age at first childbirth. All variables adjusted for except age were regarded as categorical. The continuous non-linear effect of age [25] was represented by a cubic polynomial.

In a minor subset of the data, information about potential confounders was not available, and analyses with comprehensive adjustment were based on a lower number of women than purely age-adjusted analyses. In particular, HADS scores could not be determined among 1833 women (9.3%) of the 19,637 women included in the cross-sectional adjusted analysis of associations with use of OC. Moreover, HADS scores were not available for 883 (9.7%) of the 9113 women in the adjusted cohort analysis. As HADS scores express essential psychological components potentially associated with risk of LBP [37], use of MHT [38] and OC [39], additional adjustment for HADS was made in particular sensitivity analyses. To facilitate comparison, similar analyses were also carried out with no adjustment for HADS including only the women with known HADS scores.

Separate tests were performed for interaction between use of MHT or OC and each factor adjusted for and between use of MHT and OC, in the main cross-sectional and cohort analyses with comprehensive adjustment.

All statistical analyses were carried out using IBM SPSS version 26 (IBM Corp., Armonk, New York).

## Results

### Use of MHT

In the data set analysed with respect to use of systemic MHT, never users were younger than former and current users (Table 1). No major differences were observed in the distribution of other risk factors over the categories of MHT use. Compared to participants at end of follow-up, non-participants included a slightly greater percentage of never users of MHT and a lower percentage of current users at baseline (Table S1).

**Table 1:** Descriptive statistics of potential risk factors for LBP at baseline in HUNT2, by use of systemic MHT or OC

	**Use of systemic MHT**^a^			**Use of OC**^b^		
	**Never**	**Former**	**Current**	**Never**	**Former**	**Current**
Number of women included	8222	839	2011	7777	9695	2165
Age in HUNT2^c^ (year)	50.8	54.3	54.2	50.7	39.2	28.4
BMI^c^ (kg/m^2^)	26.5	26.7	26.2	26.6	25.4	24.6
Hard physical activity per week (hour) (%^d^)						
< 1	80	80	82	80	73	60
1–2	15	15	13	14	21	27
≥ 3	5	5	5	6	7	14
Cigarette smoking (%^d^)						
Never	39	37	37	45	38	56
Daily former	29	30	32	26	27	18
Daily current	32	34	31	29	35	27
Education (year) (%^d^)						
≤ 9	40	45	42	44	18	7
10–12	39	35	36	37	53	58
≥ 13	21	21	22	19	30	35
Age at menarche^c^ (year)	13.4	13.3	13.4	13.4	13.1	13.1
Nulliparity (%^d^)	6	5	6	13	9	46
Age at first childbirth^c,e^ (year)	23.0	22.8	22.8	23.0	23.0	22.7

*LBP* Low back pain, *HUNT* Trøndelag Health Study, *MHT* Menopausal hormone therapy, *OC* Oral contraceptives, *BMI* Body mass index

^a^Among nonpregnant women aged 40–69 years with information about chronic LBP, BMI, physical activity, education, smoking, age at menarche, nulliparity, age at first childbirth, use of MHT and OC

^b^Among women aged 20–69 years with information about chronic LBP, BMI, physical activity, education, smoking, age at menarche, nulliparity, age at first childbirth, use of MHT and OC

^c^Mean value within category of use of MHT or OC

^d^Percentages of risk factor categories within category of use of MHT or OC

^e^Among women with at least 1 child

In the cross-sectional data, the highest prevalence estimate of chronic LBP was found among former users of systemic MHT (Table 2). After adjustment for age, BMI, physical activity, education, smoking, age at menarche, nulliparity, age at first childbirth and use of OC, this category showed a 46% increase in prevalence of chronic LBP compared to never users. Women who were current MHT users when information was collected experienced a 29% increase in prevalence. In contrast, the cohort analysis revealed no essential difference in risk between former and never users of systemic MHT (Table 3), but women who were current users of MHT at baseline still experienced a 30% risk increase. Sensitivity analyses with additional adjustment for HADS scores showed slightly weaker associations between use of MHT and prevalence or risk of LBP (Table S2), in particular in the cross-sectional data.

**Table 2:** Associations in HUNT2 between use of systemic MHT and prevalence of chronic LBP in cross-sectional analysis, among nonpregnant women aged 40–69 years

	**Total number of women**^**a**^	**Number of women with chronic LBP (%)**^**a**^	**PR (95% CI) with adjustment for age only**	**PR (95% CI) with comprehensive adjustment**^**b**^
Use of systemic MHT				
Number of women included			12,974	11,072
Never	9600	2682 (27.9)	1.00 (reference)	1.00 (reference)
Former	997	425 (42.6)	1.47 (1.36–1.59)	1.46 (1.34–1.59)
Current	2377	900 (37.9)	1.30 (1.22–1.38)	1.29 (1.20–1.38)
*P* for categorical effect			< 0.001	< 0.001
Duration of use of systemic MHT (year)				
Number of women included			11,988	10,284
Never used	9600	2682 (27.9)	1.00 (reference)	1.00 (reference)
0-2^c^	1256	509 (40.5)	1.40 (1.30–1.51)	1.39 (1.28–1.50)
3–5	682	250 (36.7)	1.25 (1.12–1.39)	1.27 (1.13–1.42)
6–8	224	86 (38.4)	1.29 (1.09–1.53)	1.31 (1.10–1.56)
≥ 9	226	112 (49.6)	1.68 (1.47–1.93)	1.63 (1.40–1.89)
*P* for categorical effect			< 0.001	< 0.001
*P* for linear trend^d^			0.25	0.41
Use of systemic MHT by type in current users				
Number of women included			11,825	10,107
Never used systemic	9600	2682 (27.9)	1.00 (reference)	1.00 (reference)
Oestrogen only	554	238 (43.0)	1.46 (1.32–1.62)	1.45 (1.30–1.62)
Combination	1671	604 (36.1)	1.24 (1.15–1.34)	1.23 (1.14–1.34)
*P* for categorical effect			< 0.001	< 0.001
*P* for difference between oestrogen only and combination			0.051	0.042

*HUNT* Trøndelag Health Study, *MHT* menopausal hormone therapy, *LBP* low back pain, *PR* prevalence ratio, *CI* confidence interval, *BMI* body mass index, *OC* oral contraceptives

^a^In analysis adjusted for age only

^b^Adjustment for age, BMI, physical activity, education, smoking, age at menarche, nulliparity, age at first childbirth, use of OC

^c^Must have used systemic MHT for at least one month

^d^Among all women who had used systemic MHT for at least one month

**Table 3:** Associations between use of systemic MHT in HUNT2 and risk of chronic LBP in HUNT3 in cohort analysis, among nonpregnant women aged 40–69 years at baseline

	**Total number of women**^**a**^	**Number of women with chronic LBP (%)**^**a**^	**RR (95% CI) with adjustment for age only**	**RR (95% CI) with comprehensive adjustment**^**b**^
Use of systemic MHT				
Number of women included			6007	5330
Never	4559	904 (19.8)	1.00 (reference)	1.00 (reference)
Former	385	79 (20.5)	1.06 (0.86–1.31)	1.03 (0.83–1.29)
Current	1063	262 (24.6)	1.30 (1.14–1.47)	1.30 (1.14–1.49)
*P* for categorical effect			< 0.001	0.001
Duration of use of systemic MHT (year)				
Number of women included			5564	4955
Never used	4559	904 (19.8)	1.00 (reference)	1.00 (reference)
0-2^c^	513	106 (20.7)	1.08 (0.90–1.29)	1.13 (0.94–1.36)
3–5	321	82 (25.5)	1.36 (1.11–1.66)	1.39 (1.12–1.72)
6–8	96	26 (27.1)	1.48 (1.06–2.08)	1.45 (1.02–2.07)
≥ 9	75	26 (34.7)	1.83 (1.33–2.51)	1.84 (1.31–2.59)
*P* for categorical effect			< 0.001	0.001
*P* for linear trend^d^			0.001	0.003
Use of systemic MHT by type in current users				
Number of women included			5554	4933
Never used systemic	4559	904 (19.8)	1.00 (reference)	1.00 (reference)
Oestrogen only	216	56 (25.9)	1.37 (1.08 -1.74)	1.49 (1.16–1.91)
Combination	779	195 (25.0)	1.33 (1.16–1-54)	1.35 (1.16–1.57)
*P* for categorical effect			< 0.001	< 0.001
*P* for difference between oestrogen only and combination			0.54	0.18

*MHT* Menopausal hormone therapy, *HUNT* Trøndelag Health Study, *LBP* Low back pain, *RR* Relative risk, *CI* Confidence interval, *BMI* Body mass index, *OC* Oral contraceptives

^a^In analysis adjusted for age only

^b^Adjustment for age, BMI, physical activity, education, smoking, age at menarche, nulliparity, age at first childbirth, use of OC

^c^Must have used systemic MHT for at least one month

^d^Among all women who had used systemic MHT for at least one month

A significant interaction was observed in the cross-sectional data between age in HUNT2 and use of MHT (*p* = 0.001). In subgroup analyses carried out in broad age intervals, the overall impression that former MHT users had the highest prevalence was retained for women aged < 60 years (Table S3). However, among women aged 60–69 years current users showed the greatest prevalence. In the cohort data no interaction could be established between age at baseline in HUNT2 and use of MHT (*p* = 0.74). No statistically significant interaction was observed between MHT use and other potential risk factors.

In the cohort data a marked increase in risk of chronic LBP was found with a longer duration of systemic MHT use (Table 3). Use of MHT for a period of ≥ 9 years showed the highest risk, with an estimated 84% increase compared to never users. A definite relationship between duration of MHT use and LBP prevalence could not be demonstrated in the cross-sectional data (Table 2).

The prevalence of chronic LBP depended on type of MHT in the cross-sectional data, with a greater prevalence among women using MHT based on oestrogen only compared with MHT based on combinations of oestrogen and progestin (Table 2). This contrast was less pronounced comparing risk estimates in the cohort data (Table 3).

### Use of OC

Current OC users were substantially younger than never or former users, and a much larger percentage were nulliparous (Table 1). Few former OC users were nulliparous. The lowest mean value of BMI was found among current OC users. Current users also participated in more hard physical activity and showed the lowest percentages of daily smoking. Never users of OC tended to have a considerably shorter duration of education than former and current users.

The group of women who did not participate at end of follow-up included a greater percentage of current OC users at baseline, compared to participants (Table S1). Non-participants were also a little younger and had a slightly lower percentage of higher education, but did not differ essentially in BMI, physical activity or age at menarche or first childbirth.

Adjusted estimates of relative prevalence or risk comparing overall categories of OC use were rather similar in the cross-sectional and cohort data (Tables 4 and 5). After comprehensive adjustment, former OC users showed a 17% increase in prevalence and risk compared with never users. No definite increase could be established among current users of OC. Sensitivity analyses with additional adjustment for HADS scores (Table S4) revealed only minor changes in risk estimates.

**Table 4:** Associations in HUNT2 between use of OC and prevalence of chronic LBP in cross-sectional analysis, among women aged 20–69 years

	**Total number of women**^**a**^	**Number of women with chronic LBP (%)**^**a**^	**PR (95% CI) with adjustment for age only**	**PR (95% CI) with comprehensive adjustment**^**b**^
Use of OC				
Number of women included			23,593	19,637
Never	10,441	2971 (28.5)	1.00 (reference)	1.00 (reference)
Former	10,771	2776 (25.8)	1.16 (1.11–1.23)	1.17 (1.10–1.23)
Current	2381	338 (14.2)	0.96 (0.86–1.08)	1.01 (0.89–1.15)
*P* for categorical effect			< 0.001	< 0.001
Duration of use of OC (year)				
Number of women included			23,060	19,222
0	10,441	2971 (28.5)	1.00 (reference)	1.00 (reference)
0-4^c^	6402	1576 (24.6)	1.18 (1.11–1.25)	1.16 (1.09–1.24)
5–9	3786	768 (20.3)	1.08 (1.00–1.17)	1.11 (1.03–1.21)
10–14	1728	401 (23.2)	1.09 (0.99–1.20)	1.09 (0.98–1.20)
≥ 15	703	200 (28.4)	1.16 (1.03–1.31)	1.18 (1.04–1.34)
*P* for categorical effect			< 0.001	< 0.001
*P* for linear trend^d^			0.09	0.35

*HUNT* Trøndelag Health Study, *OC* Oral contraceptives, *LBP* Low back pain, *PR* Prevalence ratio, *CI* Confidence interval, *MHT* Menopausal hormone therapy

^a^In analysis adjusted for age only

^b^Adjustment for age, BMI, physical activity, education, smoking, age at menarche, nulliparity, age at first childbirth, use of systemic MHT

^c^Must have used OC for at least one month

^d^Among all women who had used OC for at least one month

**Table 5:** Associations between use of OC in HUNT2 and risk of chronic LBP in HUNT3 in cohort analysis, among women aged 20–69 years at baseline

	**Total number of women**^**a**^	**Number of women with chronic LBP (%)**^a^	**RR (95% CI) with adjustment for age only**	**RR (95% CI) with comprehensive adjustment**^b^
Use of OC				
Number of women included			10,586	9113
Never	4523	869 (19.2)	1.00 (reference)	1.00 (reference)
Former	5066	1049 (20.7)	1.15 (1.05–1.27)	1.17 (1.06–1.30)
Current	997	166 (16.6)	1.03 (0.86–1.23)	1.07 (0.88–1.29)
*P* for categorical effect			0.010	0.008
Duration of use of OC (year)				
Number of women included			10,322	8899
0	4523	869 (19.2)	1.00 (reference)	1.00 (reference)
0-4^c^	2833	571 (20.2)	1.16 (1.04–1.30)	1.18 (1.05–1.33)
5–9	1794	357 (19.9)	1.18 (1.04–1.35)	1.22 (1.06–1.40)
10–14	833	172 (20.6)	1.17 (1.00–1.38)	1.20 (1.02–1.41)
≥ 15	339	63 (18.6)	1.02 (0.80–1.29)	1.02 (0.80–1.30)
*P* for categorical effect			0.039	0.021
*P* for linear trend^d^			0.56	0.54

*OC* Oral contraceptives, *HUNT* Trøndelag Health Study, *LBP* Low back pain, *RR* Relative risk, *CI* Confidence interval, *MHT* Menopausal hormone therapy

^a^In analysis adjusted for age only

^b^Adjustment for age, BMI, physical activity, education, smoking, age at menarche, nulliparity, age at first childbirth, use of systemic MHT

^c^Must have used OC for at least one month

^d^Among all women who had used OC for at least one month

A significant interaction between OC and BMI was found in the cross-sectional analysis (*p* = 0.044). Current OC users showed a 42% increase in prevalence of chronic LBP in the relatively small category with BMI ≥ 30 (Table S5). Current OC users with lower BMI did not exhibit any increase at all in prevalence compared to never users. In the cohort analysis, no interaction could be demonstrated between BMI and OC use (*p* = 0.15).

Duration of OC use did not appear to affect estimates of prevalence or risk of chronic LBP among the women who had used OC for at least one month (Tables 4 and 5). Estimates for separate categories of duration were still compatible with a moderate increase compared to never users of OC.

## Discussion

In this study, former users of MHT showed the highest prevalence of chronic LBP in the cross-sectional analysis. In the analysis of the cohort data 11 years after collection of information on risk factors, the former users were no longer at an increased risk of chronic LBP, but women classified as current MHT users at baseline retained a higher risk. In general, the risk increased with the number of years MHT had been used. Among former OC users, a slight increase in risk of chronic LBP was observed. Little evidence was found showing that women who were current OC users at baseline had any increased risk.

### Strengths and limitations of the study

This study was based on data obtained from an entire county in Norway. The population belonged predominantly to a homogeneous ethnic group with small socioeconomic differences [20]. The study design made it possible to carry out cross-sectional as well as cohort analyses. In both cases the information on potential risk factors related to the same period at the start of follow-up, or, in particular for MHT or OC use, to earlier time intervals. For etiologic inference the cohort results are still the most relevant, although new information on potential risk factors or LBP was not collected in the intervening period between the two surveys. No information was available on intensity of LBP.

Most of the risk factor information was based on self-reports, but height and weight were measured by trained nurses at the clinical consultation. Studies in other Scandinavian countries comparing self-reported information on use of MHT [40] or OC [41] with prescription or pharmacy data indicate that self-reports are quite accurate. To a large extent this is also the case for information on type of MHT and duration of use [40]. In the HUNT2 survey, recall bias affecting former use of MHT or OC has probably not played any major role, as respondents were unlikely to associate these factors with LBP among the large number of medical conditions dealt with in the questionnaire.

Moderate participation rates were attained in both the cross-sectional and cohort studies. Loss to follow-up occurred because of deaths, movement out of the county and final non-response. The last category was by far the largest one. The distribution of relevant baseline variables differed to some extent between respondents and non-respondents at end of follow-up, even for use of MHT or OC. Yet the two groups were not very dissimilar in overall characteristics, and there is no particular reason to assume that differential participation has seriously influenced associations with risk of chronic LBP.

The comprehensive adjustment for other potential risk factors carried out in the statistical analysis makes it unlikely that the associations observed are due to confounding. Adjustment for baseline age was especially important, in particular for associations with OC, because of strong relationships between age and both current OC use and LBP risk. Otherwise, no substantial changes in risk estimates were seen after adjustment for relevant variables, including the HADS score representing psychological factors. No adjustment was made for age at menopause, as this variable was not associated with occurrence of chronic LBP in the data set [19]. A partial adjustment for social class was performed by adjusting for duration of education, but it is still possible that some residual confounding remains.

### Previous studies

#### Use of MHT

Only two large epidemiological studies have previously dealt with associations between MHT and back pain. The study of Musgrave et al. [7] comprised 7209 women in USA aged ≥ 65 years. Analyses were carried out using both cross-sectional and cohort data, with an average follow-up of 3.7 years. The study included back pain of any kind but also applied more restrictive definitions of the disorder based on clinical significance. In most cases analysed, women reporting current oestrogen replacement therapy had the largest risk of back pain, followed by former and never users. Odds ratios as estimates of relative risk were generally of the same order of magnitude as the estimates found in the present study. However, in contrast to the results in this study, duration of use was not associated with back pain.

The cross-sectional study of Wijnhoven et al. [3] in the Netherlands included approximately 6500 women in the age range 40–59 years in analyses of associations with MHT. After adjustment for a number of relevant risk factors, women who had ever used oestrogen or female hormones because of menopausal complaints showed an increased prevalence of chronic LBP. Results were given separately for LBP with and without upper extremity pain, but in both cases the estimates were roughly consistent with those found in the present study.

Three smaller epidemiological studies, two from Sweden [6, 8] and one from the Netherlands [9], were based on different study designs. They did not produce definite results regarding associations between use of MHT and risk of back pain, although positive associations were indicated in particular cases. In a controlled trial in Finland including 48 osteopenic women randomised to oestrogen-progestin treatment or placebo, the group with hormone therapy experienced less back pain in nighttime over a 24-month follow-up period [10].

#### Use of OC

Very few large studies have considered associations between OC use and risk of back pain. The largest one to date is the cohort study of Vessey et al. [13], including 17,032 women in the United Kingdom. Participants were 25–39 years old at baseline and were followed for periods up to 26 years. Relative risks were computed for hospital referral for various back disorders, with the largest category corresponding to unspecified backache. The main conclusion was that the study did not demonstrate associations between OC use and back disorders. However, the risk estimate quoted for unspecified backache considering OC use in the past was quite similar to the estimates for LBP obtained in the present study, reflecting a very weak positive relationship.

Analyses involving OC use performed in the cross-sectional study of Wijnhoven et al. [3] included 11,428 women in the age interval 20–59 years. Current OC users had rather similar risk estimates for chronic LBP as in the present study, although risk estimates for ever users of OC were slightly greater. Women who had used OC for more than 15 years had a larger risk than those with shorter periods of use.

Various smaller epidemiological studies have dealt with relationships between OC use and LBP in cross-sectional [6, 8, 14] or longitudinal [12, 15] designs. Only one study [12] produced a definite association, with LBP occurring more frequently among OC users. Another cross-sectional study [9] found an association between OC use and prevalence of recurrent back pain.

Particular smaller studies have focused on back pain in connection with pregnancies, taking into account OC use in earlier periods. As it is difficult to distinguish between back pain and pelvic pain during pregnancy, the two disorders were combined in the relevant studies. One study [16] found essentially no relationship between such pain and OC use. Another study [17] indicated a lower risk of back pain among women who had used combination OC for > 10 years than among those with ≤ 5 years of use or no use.

### Interpretation

Despite associations found at baseline in the present study between prevalence of chronic LBP and both current and former use of MHT, the association with former use had disappeared 11 years later by the end of follow-up. These results are consistent with a general increase in risk of LBP triggered by MHT which is maintained for a certain number of years after MHT termination but then rapidly declines. Such an effect may also explain the interaction with age observed in the cross-sectional analysis. Most women aged ≥ 60 years at baseline and classified as former MHT users had most likely not used MHT for a relatively long time and would not be expected to carry any increased risk. The separate risk estimates in the cohort analysis, displaying a gradient according to duration of use, lend additional support to the hypothesis that there is a causal relationship between MHT use and risk of LBP.

Major changes in use of MHT occurred worldwide during the follow-up period in this cohort study. After results were published in 2002 indicating unfavourable associations between MHT and risk of coronary heart disease and breast cancer [42], the number of systemic MHT users in Norway dropped by about two thirds [43]. It is thus likely that a large proportion of the women in the present cohort study reporting current MHT use at baseline stopped using MHT during the follow-up period. This may have reduced the strength of the positive relationship observed between MHT and risk of chronic LBP in the cohort analysis.

For OC use no essential differences were found in the current study between cross-sectional and cohort results. The lack of any clear relationship with duration of OC use makes it more uncertain whether any causal link exists with LBP, despite the minor contrasts observed between users and non-users.

Relationships with prevalence or risk of LBP have previously been found for prior pregnancies in a woman’s life, with an increased risk in particular among women with an early first pregnancy [3, 18]. Such relationships have mainly been explained in terms of higher hormone levels affecting soft tissues supporting the spine [44] and leading to long-lasting laxity of joints and ligaments [4]. Oestrogen may play a special role in this process, possibly with the hormone relaxin as an intermediate link [45]. An increased long-term exposure to oestrogen has also been regarded as an explanation of the higher risk of LBP observed among women with an early menarche [19, 46].

Low oestrogen levels may be associated with a reduced bone mineral density (BMD) and constitute a risk factor for osteoporosis [47]. Use of MHT has for a long time been regarded as a preventive measure against osteoporosis [47]. In this way it might be conjectured that MHT use should have a protective effect in relation to musculoskeletal pain rather than being a risk factor. However, in the study of Musgrave et al. [7], use of MHT showed a positive association with BMD and an inverse association with vertebral fractures, but at the same time a positive association with occurrence of back pain. Thus the overall relationship between MHT use and LBP may be determined by other aspects of the relevant hormonal factors than those affecting BMD.

The associations between use of MHT or OC and occurrence of LBP may also be related to effects of oestrogen on pain transmission and modulation [48], but it is not evident what the influence would be on risk of LBP [49]. Postmenopausal women who receive MHT may have lower pain thresholds and tolerances than non-users [50]. More direct general effects of oestrogen include better functioning of muscles but also adverse stiffness of connective tissue [51]. While oestrogen promotes increased pain in some musculoskeletal disorders and ameliorates pain in others [52], progesterone seems to play a moderate role in reducing pain in certain musculoskeletal pain conditions [52]. This is consistent with the results from the cross-sectional part of the present work, with a larger prevalence of chronic LBP for use of systemic MHT based on oestrogen only than for combinations of oestrogen and progestin.

The data on OC use considered in this study must represent a mixture of combination OC containing both oestrogen and progestin and OC with progestin only. However, at the time when follow-up was completed, progestin-only OC constituted a relatively minor proportion of the total OC use in Norway [53], and when risk factor information was collected and before, the proportion of progestin-only users must have been even smaller. Thus the weak associations observed between OC use and chronic LBP, if real, are most likely directly related to oestrogen, although the amount of oestrogen included in OC has declined over time [54]. Adipose tissue produces an array of hormones that may play a role in metabolic homeostasis [55], with possible differences in the handling of steroid hormones depending on body mass [56]. This may account for the interaction indicated between OC use and BMI in the cross-sectional data.

If the associations found in this work are related to the oestrogen contents of MHT and OC, the results are likely to apply to other populations as well, despite minor differences in the composition of MHT or OC. The increase suggested in risk of chronic LBP with OC use is in any case of less practical importance than the implications of MHT use. According to the basic cohort analysis, use of MHT among women in the relevant age group may increase the absolute risk of chronic LBP among unaffected women from about 20% to 26%. Long-term use might lead to considerably larger absolute risks. Present recommendations regarding use of MHT are rather complex, taking into account potential effects on various medical conditions but not back pain [57]. It may be reasonable to modify these recommendations if more information becomes available on relationships with other conditions such as LBP.

## Conclusions

Use of systemic MHT appears to be associated with an increased risk of chronic LBP, during the period of use and for some time afterwards. The risk increases with duration of use. The risk may be somewhat greater for MHT with oestrogen only than for MHT including both oestrogen and progestin. Use of OC may possibly be associated with a small increase in risk of chronic LBP.

## Supplementary Information

**Additional file 1:** **Table S1.** Descriptive statistics of potential risk factors for LBP at baseline in HUNT2, by follow-up status. **Table S2.** Associations between use of systemic MHT and prevalence or risk of chronic LBP in cross-sectional and cohort analysis, among nonpregnant women aged 40-69 years, with and without adjustment for HADS. **Table S3.** Associations between use of systemic MHT and prevalence or risk of chronic LBP in cross-sectional and cohort analysis, among nonpregnant women aged 40-69 years, in broad intervals of age in HUNT2. **Table S4.** Associations between use of OC and prevalence or risk of chronic LBP in cross-sectional and cohort analysis, among women aged 20-69 years, with and without adjustment for HADS. **Table S5.** Associations between use of OC and prevalence or risk of chronic LBP in cross-sectional and cohort analysis, among women aged 20-69 years, in intervals of BMI in HUNT2.

## Data Availability

The data set analysed belongs to a third party, the HUNT Study (the Trøndelag Health Study). The authors of the current manuscript have been given permission to analyse the data after obtaining the necessary Norwegian permits. Because of the confidentiality requirements, a data set of this kind with information at the individual level cannot be made public. However, research groups who want to analyse data from the HUNT Study may apply to the HUNT organisation (https://www.ntnu.edu/hunt) to get access, having obtained the permits needed according to Norwegian law.

## Abbreviations

MHT: Menopausal hormone therapy
OC: Oral contraceptives
BMI: Body mass index
CI: Confidence interval
HADS: Hospital anxiety and depression scale
HUNT: Trøndelag Health Study
LBP: Low back pain
PR: Prevalence ratio
RR: Relative risk
