# Supplementary material for: Menopausal hormone therapy, oral contraceptives and risk of chronic low back pain: the HUNT Study
Source: BMC Musculoskelet Disord. 2023 Jan 31;24:84. doi: 10.1186/s12891-023-06184-5 (PMC9887847; doi:10.1186/s12891-023-06184-5)
Supplement: Supplementary file 1 — Additional file 1: Table S1. Descriptive statistics of potential risk factors for LBP at baseline in HUNT2, by follow-up status. Table S2. Associations between use of systemic MHT and prevalence or risk of chronic LBP in cross-sectional and cohort analysis, among nonpregnant women aged 40-69 years, with and without adjustment for HADS. Table S3. Associations between use of systemic MHT and prevalence or risk of chronic LBP in cross-sectional and cohort analysis, among nonpregnant women aged 40-69 years, in broad intervals of age in HUNT2. Table S4. Associations between use of OC and prevalence or risk of chronic LBP in cross-sectional and cohort analysis, among women aged 20-69 years, with and without adjustment for HADS. Table S5. Associations between use of OC and prevalence or risk of chronic LBP in cross-sectional and cohort analysis, among women aged 20-69 years, in intervals of BMI in HUNT2. [file 12891_2023_6184_MOESM1_ESM.pdf]

**Table S1** Descriptive statistics of potential risk factors for LBP at baseline in HUNT2, by follow-up status

| | **Died during follow-up**[a] | **Left the county during follow-up**[a] | **Non-participants at end of follow-up**[a] | **Participants at end of follow-up**[a] |
|---|---|---|---|---|
| Number of women included | 354 | 974 | 4296 | 9113 |
| Age in HUNT2[b] (year) | 56.3 | 31.3 | 39.1 | 43.0 |
| BMI[b] (kg/m$^2$) | 26.8 | 24.2 | 25.9 | 25.5 |
| Hard physical activity per week (hour) (%[c]) | | | | |
| < 1 | 86 | 57 | 74 | 72 |
| 1-2 | 9 | 28 | 19 | 21 |
| ≥ 3 | 5 | 16 | 7 | 7 |
| Education (year) (%[c]) | | | | |
| ≤ 9 | 55 | 8 | 25 | 24 |
| 10-12 | 31 | 45 | 51 | 47 |
| ≥ 13 | 14 | 47 | 24 | 28 |
| Age at menarche[b] (year) | 13.5 | 13.1 | 13.2 | 13.3 |
| Age at first childbirth[b,d] (year) | 23.3 | 23.4 | 22.8 | 23.3 |
| Nulliparity (%[c]) | 9 | 56 | 19 | 12 |
| Use of systemic MHT[e] (%[c]) | | | | |
| Never | 77 | 73 | 81 | 77 |
| Former | 10 | 7 | 5 | 6 |
| Current | 13 | 20 | 14 | 17 |
| Use of OC (%[c]) | | | | |
| Never | 73 | 30 | 36 | 39 |
| Former | 25 | 41 | 49 | 51 |
| Current | 2 | 28 | 16 | 10 |

*HUNT* Trøndelag Health Study, *BMI* body mass index, *MHT* menopausal hormone therapy, *OC* oral contraceptives, *LBP* low back pain

[a]Among women aged 20-69 years included in follow-up with baseline information about BMI, physical activity, education, smoking, age at menarche, nulliparity, age at first childbirth, use of MHT and OC

[b]Mean value within category of follow-up status

[c]Percentages of risk factor categories within category of follow-up status

[d]Among women with at least 1 child

[e]Among nonpregnant women aged 40-69 years

**Table S2** Associations between use of systemic MHT and prevalence or risk of chronic LBP in cross-sectional and cohort analysis, among nonpregnant women aged 40-69 years, with and without adjustment for HADS[a]

| | With standard comprehensive adjustment[b] | With additional adjustment for HADS score |
|---|---|---|
| | PR[c] or RR[d] (95% CI) | PR[c] or RR[d] (95% CI) |
| **Cross-sectional analysis** | | |
| Use of systemic MHT | | |
| Number of women included | 9537 | 9537 |
| Never | 1.00 (reference) | 1.00 (reference) |
| Former | 1.47 (1.33-1.61) | 1.34 (1.23-1.47) |
| Current | 1.26 (1.17-1.36) | 1.20 (1.11-1.29) |
| P for categorical effect | < 0.001 | < 0.001 |
| **Cohort analysis** | | |
| Use of systemic MHT | | |
| Number of women included | 4695 | 4695 |
| Never | 1.00 (reference) | 1.00 (reference) |
| Former | 1.03 (0.81-1.30) | 1.00 (0.79-1.26) |
| Current | 1.27 (1.09-1.47) | 1.24 (1.07-1.44) |
| P for categorical effect | 0.009 | 0.015 |

*MHT* menopausal hormone therapy, *LBP* low back pain, *HADS* Hospital Anxiety and Depression Scale, *PR* prevalence ratio, *RR* relative risk, *CI* confidence interval, *BMI* body mass index, *OC* oral contraceptives

[a]Among women with known HADS score

[b]Adjustment for age, BMI, physical activity, education, smoking, age at menarche, nulliparity, age at first childbirth, use of OC

[c]In cross-sectional analysis

[d]In cohort analysis

**Table S3** Associations between use of systemic MHT and prevalence or risk of chronic LBP in cross-sectional and cohort analysis, among nonpregnant women aged 40-69 years, in broad intervals of age in HUNT2

| | Age 40-49 years | Age 50-59 years | Age 60-69 years |
|---|---|---|---|
| | **PR[a] or RR[b] (95% CI)[c]** | **PR[a] or RR[b] (95% CI)[c]** | **PR[a] or RR[b] (95% CI)[c]** |
| **Cross-sectional analysis** | | | |
| Use of systemic MHT | | | |
| Number of women included | 5323 | 3715 | 2034 |
| Never | 1.00 (reference) | 1.00 (reference) | 1.00 (reference) |
| Former | 1.55 (1.35-1.78) | 1.53 (1.37-1.72) | 1.19 (0.97-1.46) |
| Current | 1.15 (1.01-1.31) | 1.28 (1.17-1.40) | 1.53 (1.34-1.74) |
| P for categorical effect | < 0.001 | < 0.001 | < 0.001 |
| **Cohort analysis** | | | |
| Use of systemic MHT | | | |
| Number of women included | 2739 | 1767 | 824 |
| Never | 1.00 (reference) | 1.00 (reference) | 1.00 (reference) |
| Former | 1.09 (0.75-1.59) | 0.89 (0.62-1.28) | 1.19 (0.79-1.78) |
| Current | 1.25 (1.00-1.57) | 1.34 (1.13-1.59) | 1.23 (0.88-1.71) |
| P for categorical effect | 0.17 | 0.003 | 0.42 |

*MHT* menopausal hormone therapy, *LBP* low back pain, *HUNT* Trøndelag Health Study, *PR* prevalence ratio, *RR* relative risk, *CI* confidence interval, *BMI* body mass index, *OC* oral contraceptives

[a]In cross-sectional analysis

[b]In cohort analysis

[c]Adjustment for age, BMI, physical activity, education, smoking, age at menarche, nulliparity, age at first childbirth, use of OC

**Table S4** Associations between use of OC and prevalence or risk of chronic LBP in cross-sectional and cohort analysis, among women aged 20-69 years, with and without adjustment for HADS[a]

| | **With comprehensive adjustment[b]** | **With additional adjustment for HADS score** |
|---|---|---|
| | **PR[c] or RR[d] (95% CI)** | **PR[c] or RR[d] (95% CI)** |
| **Cross-sectional analysis** | | |
| Use of OC | | |
| Number of women included | 17,804 | 17,804 |
| Never | 1.00 (reference) | 1.00 (reference) |
| Former | 1.18 (1.11-1.25) | 1.15 (1.09-1.22) |
| Current | 1.02 (0.90-1.16) | 1.02 (0.90-1.16) |
| P for categorical effect | < 0.001 | < 0.001 |
| **Cohort analysis** | | |
| Use of OC | | |
| Number of women included | 8230 | 8230 |
| Never | 1.00 (reference) | 1.00 (reference) |
| Former | 1.22 (1.09-1.36) | 1.22 (1.09-1.36) |
| Current | 1.10 (0.90-1.34) | 1.09 (0.90-1.33) |
| P for categorical effect | 0.001 | 0.002 |

*OC* oral contraceptives, *LBP* low back pain, *HADS* Hospital Anxiety and Depression Scale, *PR* prevalence ratio, *RR* relative risk, *CI* confidence interval, *BMI* body mass index, *MHT* menopausal hormone therapy

[a]Among women with known HADS score

[b]Adjustment for age, BMI, physical activity, education, smoking, age at menarche, nulliparity, age at first childbirth, use of systemic MHT in the cohort analysis; no adjustment for age at menarche in the cross-sectional analysis because of non-convergence with the full model

[c]In cross-sectional analysis

[d]In cohort analysis

**Table S5** Associations between use of OC and prevalence or risk of chronic LBP in cross-sectional and cohort analysis, among women aged 20-69 years, in intervals of BMI in HUNT2

| | BMI <25 ($kg/m^2$) | BMI 25-29.9 ($kg/m^2$) | BMI ≥30 ($kg/m^2$) |
|---|---|---|---|
| | PR[a] or RR[b] (95% CI)[c] | PR[a] or RR[b] (95% CI)[c] | PR[a] or RR[b] (95% CI)[c] |
| **Cross-sectional analysis** | | | |
| Use of OC | | | |
| Number of women included | 9664 | 7006 | 2967 |
| Never | 1.00 (reference) | 1.00 (reference) | 1.00 (reference) |
| Former | 1.13 (1.05-1.23) | 1.19 (1.10-1.29) | 1.17 (1.05-1.30) |
| Current | 0.90 (0.76-1.07) | 1.01 (0.82-1.23) | 1.42 (1.12-1.80) |
| P for categorical effect | 0.001 | < 0.001 | 0.002 |
| **Cohort analysis** | | | |
| Use of OC | | | |
| Number of women included | 4671 | 3251 | 1191 |
| Never | 1.00 (reference) | 1.00 (reference) | 1.00 (reference) |
| Former | 1.23 (1.06-1.43) | 1.12 (0.96-1.29) | 1.18 (0.94-1.49) |
| Current | 1.10 (0.86-1.40) | 1.18 (0.90-1.56) | 0.52 (0.24-1.13) |
| P for categorical effect | 0.017 | 0.27 | 0.030 |

*OC* oral contraceptives, *LBP* low back pain, *BMI* body mass index, *HUNT* Trøndelag Health Study, *PR* prevalence ratio, *RR* relative risk, *CI* confidence interval, *MHT* menopausal hormone therapy

[a]In cross-sectional analysis

[b]In cohort analysis

[c]Adjustment for age, physical activity, education, smoking, age at menarche, nulliparity, age at first childbirth, use of systemic MHT
